# Quantifying the Impact of Family Doctors on the Care Experiences of Patients with Cancer: Exploring Evidence from the 2021 Ambulatory Oncology Patient Satisfaction Survey in Alberta, Canada

**DOI:** 10.3390/curroncol30010049

**Published:** 2023-01-04

**Authors:** Linda Watson, Claire Link, Siwei Qi, Andrea DeIure

**Affiliations:** 1 Applied Research & Patient Experience, Cancer Research & Analytics, Cancer Care Alberta—Alberta Health Services, Calgary, AB T2S 3C3, Canada; 2 Faculty of Nursing, University of Calgary, Calgary, AB T2N 1N4, Canada

**Keywords:** ambulatory oncology patient satisfaction survey, AOPSS, patient experience, patient satisfaction, family doctor, primary care, medical home, shared care, cancer care, cancer treatment

## Abstract

Oncology programs across Canada are reaching capacity as more Canadians are diagnosed with and treated for cancer each year. There is an increasing need to share care with family doctors, however it is unclear how this type of care impacts patient experiences, particularly while receiving active treatment. Retrospective data from the 2021 Ambulatory Oncology Patient Satisfaction Survey (AOPSS) in Alberta, Canada was used in this study. A unique question on the Alberta survey asks patients about their family doctor’s involvement during their cancer care. Patient satisfaction across the six domains of person-centred care on the AOPSS was analyzed based on how involved a patient’s family doctor was. Compared to patients who indicated their family doctor was “Not involved”, patients with “Very involved” family doctors had significantly higher satisfaction scores in all six domains of care. The three domains which showed the largest positive impact of family doctor involvement were: Coordination & Integration of Care, Emotional Concerns, and Information, Communication & Education. The results demonstrate that involving family doctors in cancer care can be beneficial for patients. Based on the observed satisfaction increases in this study, shared care models may be preferred by many patients. These models of care can also help alleviate strain and capacity issues within cancer programs. The results could be used to support recommendations for cancer care teams to regularly involve and communicate with family doctors, to ensure that patients receive comprehensive and tailored care from all their health care providers.

## 1. Introduction

Undergoing cancer treatment can often be challenging for patients. They may experience a multitude of physical and psychological symptoms and side effects such as fatigue, nausea, depression, anxiety, and pain [1,2,3,4]. These side effects can be episodic or long-standing, but either way can negatively impact patients’ quality of life [5]. To feel supported during their cancer journey, patients may benefit from regular connection and consultation with multiple care providers, including their oncology team, supportive care providers, and their family doctor or other primary care provider (PCP), to ensure they receive comprehensive and coordinated care [6,7].

The role of the family doctor may shift throughout a patient’s cancer journey. Family doctors are often the first point of contact for patients when they experience symptoms that may lead to a cancer diagnosis, with a recent study suggesting that most PCPs diagnose one cancer each month [8]. Studies have found that adherence to cancer screening recommendations is higher among patients who regularly see and/or have a strong relationship with their family doctor, across multiple cancer types [9,10,11]. Once a patient is formally diagnosed and begins to receive care in a cancer program, some family doctors feel that their role becomes more ambiguous [12,13]. Many cancer programs have a highly skilled team of interdisciplinary specialists, and as a result primary care may be excluded from this phase of care, largely due to poor communication from the oncology team [12,13]. A study conducted in Israel found that, of patients receiving chemotherapy, only 30% indicated that their oncologist communicated with their family doctor [14]. Similarly, a Canadian study found that less than 45% of primary care providers were involved at the treatment phase of their patient’s lung cancer journey [15]. Patients may also decrease the frequency of visits to their family doctor while undergoing cancer treatment, choosing to bring all health concerns to their oncology team, and returning to more frequent visits with their family doctor after active treatment is complete [15]. It is at this point in the cancer journey that the family doctor role can become clearer again, as patients may be transitioned out of cancer care to have follow-up and surveillance activities performed in primary care settings [16]. However, a continued shortage of communication between the cancer program and primary care may lead to a lack of role clarity regarding which provider will manage different aspects of follow-up care [17,18].

As the rates of cancer incidence continue to rise, oncology programs across the country are reaching capacity. There is a critical need to collaborate with primary care to share the care of cancer patients, to help manage the increasing workload in cancer care and ensure there is capacity to continue seeing newly diagnosed patients [13,14]. Shared models of care are being explored and/or tested in multiple countries, with most focusing on the post-treatment follow-up phase of the cancer journey, with varying levels of success [15,19,20,21]. In Canada, it is currently unclear how often shared care models are utilized, particularly when patients are undergoing active treatment. Highlighting the positive impacts of family doctor involvement in routine cancer care may help make a stronger case for why they should be included as a key member of the care team throughout a patient’s cancer care journey. In the province of Alberta, Canada, the provincial cancer program, Cancer Care Alberta (CCA), has been using a routine patient experience survey for many years, which has recently included a unique question about the level of involvement of a patient’s family doctor during their cancer care. Our aim was to use retrospective survey data to understand how family doctor involvement may be associated with patients’ satisfaction with their cancer care, specifically during active treatment.

## 2. Materials and Methods

### 2.1. Study Design

For this study we used retrospective cross-sectional data from the Ambulatory Oncology Patient Satisfaction Survey (AOPSS) in Alberta [22,23]. This survey is distributed semi-annually to a random selection of patients who have received cancer treatment(s) in the last six months at one of the 17 ambulatory cancer centres in CCA. The data in this study were from the most recent survey cycle, collected between February and late May of 2021. The AOPSS was administered in compliance with the Declaration of Helsinki and the Alberta Research Ethics Community Consensus Initiative (ARECCI) ethical guidelines for quality improvement and evaluation [24]. Based on the results of the ARECCI screening, the survey was classified as a quality improvement initiative and full ethics review was not required. No harm was anticipated or reported in relation to this survey.

### 2.2. Study Setting and Sample

In the province of Alberta, cancer care is publicly funded and delivered primarily through Cancer Care Alberta, a provincial program made up of 17 cancer centres which span the province [25]. There are two large tertiary cancer centres located in major cities, four regional centres located in smaller cities, and eleven community centres, co-located in select small city or town hospitals. All centres offer ambulatory cancer services, including treatment (although some treatment options are only available at certain sites), however the community sites are only able to administer a treatment plan which has been ordered by an oncologist at either a tertiary or regional centre [25].

The AOPSS was distributed to a random sample of Albertans who had received ambulatory cancer treatment at one of the 17 cancer centres in the previous six months. The AOPSS defines treatment as surgery, cancer medications (IV chemotherapy, immunotherapy, and oral chemotherapy) and radiation therapy [23]. In CCA, surgery is typically performed outside of the cancer program; because of this, surgery-only patients were excluded from the sample. While the sample included patients treated at all 17 cancer centres, there was greater representation from the two tertiary centres. To simplify the analysis, we grouped the regional and community cancer centres together, and grouped the two tertiary centres together as well.

### 2.3. Survey Procedure

Planning began in August of 2020, with survey distribution, involving mailing paper copies of the survey and including a pre-paid return envelope, occurring in February 2021. In each survey package there was also a link to an online version of the survey and a unique patient identifier code. In total, 4000 surveys were distributed, 39 of which (1.0%) were non-deliverable [26]. A reminder letter was sent several weeks after the initial mail-out to patients who had not yet responded, and the survey officially closed in late May.

### 2.4. Measures

The AOPSS was developed by the National Research Corporation Canada (NRC) in 2003, based on the existing US Picker Cancer Care survey [23]. This existing survey was modified to fit the context of ambulatory cancer care in Canada [27,28]. The AOPSS consists of forty-four core survey questions used to construct six domains of person-centred care. These domains are then used to categorize and understand patient experiences. The six domains are “Access to Care”, “Coordination & Integration of Care”, “Emotional Support”, “Information, Communication & Education”, “Physical Comfort”, and “Respect for Patient Preferences”. Domain scores represent an average of the responses to specific questions under each domain and are transformed linearly to a 0%-to-100% scale following scoring guidance from NRC [22]. Not all domains have an equal number of questions. There are multiple other questions on the survey that do not fall into one of the domains, however these questions were not the focus of this study. The survey contains one question that assesses overall patient experience and satisfaction with cancer care: “Overall, how would you rate the quality of all of your care in the past 6 months?” This question, along with the six domain scores, were analyzed as the outcomes in this study.

The AOPSS includes one question about family doctors, specifically: “If you had a visit with your family doctor in the past 6 months, did you feel your family doctor knew enough about your cancer care?”. The response options are “Yes, completely”, “Yes, somewhat”, “No”, and “Doesn’t apply”. However, we did not feel that this question was the best way to quantify the impact of a family doctor on a patient’s experience, due to the vagueness of the wording and the focus on family doctor awareness rather than patient care. In the most recent survey cycle we had the option of adding five unique questions to the Alberta version of the AOPSS. To understand how family doctor involvement may impact patient experiences during their cancer treatment, we chose to add an additional question to CCA’s version of the 2021 AOPSS, which asked respondents: “How involved has your family doctor been in your care while you have been on cancer treatments?”. There were six response options: “Very involved”, “Somewhat involved”, “Not very involved”, “Not at all involved”, “I do not have a family doctor”, and “Unsure”. To attain a more balanced distribution, the levels of “Not very involved” and “Not at all involved” were collapsed into one group (“Not involved”).

To control for potential confounding factors, several sociodemographic and clinical characteristics were included. Specifically, we looked at age group, sex, education, tumour group, cancer centre visited (the centre where the patient was treated—tertiary or regional/community), time since diagnosis (in months), and treatment intent. Treatment intent was measured using another question that is unique to CCA’s version of the AOPSS, which asks patients if the intent of their treatment was to cure their cancer or control their symptoms and/or disease progression. Details of this question have been reported previously by the authors [26].

The final question on the AOPSS is open-ended, and asks, “Is there anything else you would like to tell us about your cancer care services?”. To explore whether respondents made any reference to family doctors in their comments, we performed a keyword search using the following terms: family doctor, family Dr, family md, family physician, GP, general practitioner, and primary care. Once relevant comments were identified, they were grouped into overall themes and/or categories.

### 2.5. Statistical Analyses

Demographic and clinical characteristics were examined for the whole sample and were also cross-tabulated by the level of family doctor involvement and compared to assess statistically significant differences, using chi-squared tests for categorical variables and Analysis of Variance (ANOVA) for continuous variables. To compare patient experiences by the level of family doctor involvement, multiple linear regressions were utilized, with each of the six domain scores constructed as a linear outcome. One domain score was selected as the dependent variable in each of the six models, with family doctor involvement being the primary association of interest. For each model, the “Not involved” category was set as the reference group, and baseline demographic and clinical characteristics were controlled for. To assess the difference in overall satisfaction by the level of family doctor involvement, multivariate ordinal logistic regressions were utilized for the overall satisfaction question, again controlling for baseline and clinical characteristics. For analysis, SPSS software was used (Version 25.0), with statistical significance set a prior at *p* < 0.05.

## 3. Results

### 3.1. Sample Characteristics

The response rate for the survey was 55.6%, with 2204 surveys returned. Most surveys were completed on paper and returned by mail, with very few respondents completing the survey online. Of the 2204 respondents who completed the survey, 1212 (55.0%) were women and 992 were men (45.0%). The mean age of the sample was 66.5 (SD = 11.7), with more than half of respondents (61.6%) aged 65 or older. More than half had post-secondary education (57.1%) and 57.8% of respondents received treatment in a tertiary cancer centre. The most common tumour group in the study sample was hematological (26.4%), followed by breast (22.3%) and gastrointestinal (14.6%). The average time since diagnosis was 39.7 months (SD = 54.7) and just over half of the respondents (51.1%) indicated that the goal of their treatment was non-curative and was intended to control, rather than cure, their cancer. All sociodemographic and clinical characteristics for the full sample are presented in Table 1.

### 3.2. Family Doctor Involvement and Patient Characteristics

Most respondents (*n* = 2102, 95.4%) answered the question regarding family doctor involvement and 102 (4.6%) skipped this question. Of the 2102 respondents who answered this question, 41 (2.0%) chose “I do not have a family doctor”, and 82 (3.9%) chose “Unsure”, both of which were excluded from the data analysis, leaving a total of 1979 respondents.

Just over one-quarter of respondents indicated they had a family doctor who was “Very involved” (*n* = 506, 25.6%), one-third indicated they had a family doctor who was “Somewhat involved” (*n* = 656, 33.1%) and 41.3% indicated that their family doctor was “Not involved” (*n* = 817). Table 1 presents the distribution of sociodemographic and clinical characteristics for each of the three groups. Family doctor involvement was significantly associated with age group, χ^2^ (8, *n* = 1979) = 15.6, *p* < 0.05; level of education, χ^2^ (4, *n* = 1902) = 25.2, *p* < 0.01; tumour group, χ^2^ (12, ***n*** = 1979) = 21.3, *p* < 0.05; treatment intent, χ^2^ (2, *n* = 1864) = 6.44, *p* < 0.05; and cancer centre where the patient received treatment, χ^2^ (2, *n* = 1979) = 11.6, *p* < 0.01. No significant association was noted between family doctor involvement and sex (*p* > 0.05) or time since diagnosis (*p* > 0.05).

### 3.3. Family Doctor Involvement and Six Dimensions of Person-Centred Care

Multiple linear regression was used to examine the association between family doctor involvement and the six dimensions of person-centred care on the AOPSS, while controlling for age, sex, education level, tumour group, treatment intent, location of care, and time since diagnosis. In the first model, the domain score for “Access to Care” was the dependent variable, with family doctor involvement as the association of interest. A significant *F*-test [F (6) = 83.1, *p* < 0.01] indicated that the proposed association between the outcome and the set of predictors was statistically reliable. Family doctor involvement was found to be significantly associated with “Access to Care” scores (*p* < 0.01), after controlling for other potentially confounding variables. Specifically, patients who indicated that their family doctor was “Very involved” in their care were significantly more likely to report higher satisfaction scores than patients whose family doctor was “Not involved” (t = 3.30, *p* < 0.01) in their care. Patients who indicated that their family doctor was “Somewhat involved” in their care were also more likely to report higher satisfaction scores, however this difference did not reach statistical significance (t = 1.77, *p* = 0.078).

Similar regression models of the same structure were performed to assess the association between family doctor involvement and the other five domain scores. Compared with patients whose family doctor was “Not involved”, patients whose family doctor was “Very involved” reported significantly higher scores, on average, for “Coordination & Integration of Care” (t = 10.3, *p* < 0.001), “Emotional Support” (t = 8.64, *p* < 0.001), “Information, Communication & Education” (t = 8.21, *p* < 0.001), “Physical Comfort” (t = 2.77, *p* < 0.01) and “Respect for Patient Preferences” (t = 5.47, *p* < 0.001). Patients with a “Somewhat involved” family doctor were also more likely to report significantly higher satisfaction scores for “Coordination & Integration of Care” (t = 5.02, *p* < 0.001), “Emotional Support” (t = 4.66, *p* < 0.001), “Information, Communication & Education” (t = 3.93, *p* < 0.001) and “Respect for Patient Preferences” (t = 3.86, *p* < 0.01). Scores did not significantly differ for “Physical Comfort” between patients with a “Somewhat involved” and a “Not involved” family doctor. Details of the parameter estimates for each model are presented in Table 2.

### 3.4. Family Doctor Involvement and Overall Satisfaction with Care

Using the overall quality of care question as the outcome in the ordinal logistic regression model, while controlling for sociodemographic and clinical characteristics, we examined the association between family doctor involvement and overall patient satisfaction. Results from the ordinal logistic regression showed that, after adjusting for the confounders, patients with a “Very involved” family doctor had 2.31 (1.80–2.96) times higher odds of reporting higher overall satisfaction with the quality of their care compared to patients whose family doctor was “Not involved” (*p* < 0.001). Similarly, patients whose family doctor was “Somewhat involved” had 1.32 (1.07–1.62) times higher odds of reporting higher overall satisfaction with their quality of care (*p* < 0.05). Details of the model are presented in Table 3.

### 3.5. Open-Ended Responses Regarding Family Doctor Involvement

In total, of the 2204 respondents, 891 left an open-ended comment (40.4%). Of these, the keyword search returned 44 relevant comments (4.9%) which referred to family doctor(s) in some way. After reviewing the comments, one was excluded as it referenced a family member as a “primary caregiver”, rather than discussing a PCP such as a family doctor. Of the remaining 43 comments, ten (23.3%) mentioned family doctors briefly, however the overall comments were not about family doctors or their involvement in care. Only select open-ended results are shared here; please contact the corresponding author for additional results. There were 15 comments (34.9%) praising the excellent care provided by both the oncology team and the patient’s family doctor. Several comments exemplify this, such as “I feel I have a great team, from the family physician and my oncologist” and “My overall care was fantastic, appreciate everything from my family doctor, my oncologist, [and] doctors at the cancer centre.” A small number of comments expressed dissatisfaction with the level of involvement their family doctor had in their cancer care. This was evidenced in comments like “We kept contact with my family doctor but he would not offer opinions or advice” and “I would like to have a family doctor who would be able, at this time, to be more in charge of my care”. Other patients felt there was a gap in communication between the oncology team and their family doctor, noting that “Results were not on my family doctor’s computer” and “My biggest concern is the lack of communication my family doctor receives about my cancer treatment.”

## 4. Discussion

This study emphasizes the important role that family doctors play in the ongoing care of patients with cancer, especially while undergoing treatment. The findings of this study help quantify the impact that family doctors can have on patient experiences and satisfaction and suggest that cancer care teams should try to involve family doctors in patients’ care. The “family doctor involvement” question is unique to the AOPSS administered in Alberta, however we recommend that other provinces and organizations who use the AOPSS as a patient experience measure consider adding this question as well. It can be difficult to measure the impact of something like family doctor involvement, which is indefinable in many ways, however this question provides a method for achieving this. Positive findings like these may help in promoting the importance of “shared care” models for patients with cancer [13], as patients who had an involved family physician while they were receiving cancer treatment were significantly more satisfied with all aspects of their care.

The results showed that patients with a “Very involved” family doctor had significantly higher satisfaction in all six domains of care, and patients with a “Somewhat involved” family doctor had higher satisfaction in four domains, compared to patients who did not have an involved family doctor. Both groups also had significantly higher odds of rating the overall quality of their care more positively, with the “Very involved” group’s odds more than twice as high as the “Not involved” group. It is interesting to note that nearly 5% of the open-ended comments referred to family doctors in some way. Given that this question is entirely free-text based, with no mention of family doctors as a prompt, this highlights that patients also feel that the role of the family doctor is relevant to their cancer care journey. The three domains in which family doctor involvement had the largest positive impact were Coordination & Integration of Care, Emotional Concerns, and Information, Communication & Education.

### 4.1. Coordination & Integration of Care

Coordination and integration of care was the domain that showed the largest positive impact of having a “Very involved” family doctor, and the second-largest positive impact of having a “Somewhat involved” family doctor. This may be due to the likelihood that successful integration and coordination of care requires effective communication between the cancer care team and family doctor. If a family doctor is regularly receiving updates from the cancer care team regarding a patient’s treatments, progress, and symptoms, they are in turn better equipped to provide support to that patient and effectively manage their concerns as needed [18]. In contrast, if a family doctor does not receive updates or information from the cancer care team, it makes it difficult for them to be fully involved in supporting their patient, as they do not have a clear understanding of the patient’s needs and experiences on an ongoing basis.

### 4.2. Emotional Concerns

The “emotional concerns” domain showed the largest positive impact of having a “Somewhat involved” family doctor and the second-largest impact of having a “Very involved” family doctor. Some patients feel that their psychosocial needs are not adequately met when receiving care in specialist programs such as oncology care [29]. Many family doctors regularly provide considerable emotional support as part of their typical practice, with one study reporting that 60% of family doctors stated they were the sole provider responsible for managing anxiety and depression as part of cancer follow-up care [30]. Another Canadian study found that patients, cancer specialists, and primary care providers all agreed that primary care should provide emotional support throughout all phases of the cancer journey [15]. Family practices have been described as a patient’s “medical home”, as the PCPs there have the most extensive knowledge of a patient’s background, including both their medical and social history [31,32]. This enables them to provide emotional support that may be more tailored to each individual patient, compared to support provided by a specialist with limited knowledge about a patient’s background. A patient may also feel more comfortable or trusting of their family doctor, perhaps especially if they have been seeing the same doctor for many years, which may also contribute to the higher satisfaction with their emotional needs being met. Emotional support has previously been identified as an aspect of care that could be improved upon in CCA [33] and across other provincial cancer jurisdictions as well [28,34,35,36]. A study conducted in Ireland found that breast cancer patients receiving chemotherapy were least satisfied with the emotional support they received, as well as the amount of information they received [37] (this latter point will be explored further). If family doctors can help fill this gap by providing more emotional support, patient satisfaction and experiences may improve universally.

### 4.3. Information, Communication & Education

This domain of care showed the third-largest positive impact of both “Very involved” and “Somewhat involved” family doctors. If patients are regularly seeing their family doctor, they may have increased access to resources and educational materials, in contrast to patients who might only have access to the resources provided by the cancer care team or internal to the cancer program. A recent Canadian study found that, in a survey of cancer survivors who had completed treatment, less than 60% of respondents felt they been provided with enough information about community resources (patients treated for hematological cancers were the exception, with around 70% having enough information) [38]. More respondents agreed that they had received enough information about signs of cancer recurrence and treatment side effects. It is not unexpected that cancer care providers would have more information and resource recommendations on those topics. Informational needs in general have been highlighted as a gap or area for quality improvement in previous studies [28,38], and family doctors can play a key role in addressing this gap. Family doctors likely have a greater awareness and connection to community resources, whether services are available through their own clinic, their primary care network, or other community organizations.

### 4.4. The Importance of Sharing Care

While the positive impacts of family doctor involvement are clear in this study, it is important to recognize that more than 40% of survey respondents reported that their family doctor was not involved in their care, with almost 4% indicating they were unsure about their family doctor’s involvement and another 2% indicating they did not have a family doctor at all. This means that many patients did not experience the benefits of having an involved family doctor throughout their cancer care with CCA. This was seen in some of the open-ended comments, with some respondents indicating that their family doctor was not as involved as they would have liked them to be. This speaks to the idea that simply having a family doctor is not necessarily enough; if a patient’s doctor is not engaged and involved in their cancer care, the benefits to the patient are minimal. Oncology care providers could help ensure that more cancer patients are connected to a family doctor by emphasizing the positive benefits of this connection to their patients and providing them with resources for obtaining a family physician. For example, in Alberta there is a dedicated webpage where patients can enter an address and find family doctors in their proximity who are actively accepting new patients [39]. This webpage is featured on multiple patient-facing resources within CCA, and care providers can also promote this resource to their patients during clinic appointments. In addition, oncology care teams should encourage patients who already have a family doctor to connect with them regularly and keep them involved and up to date throughout their cancer care journey. As was highlighted in the open-ended comments, cancer care providers and teams should maintain regular communication with family doctors to ensure that when patients have a visit, their doctor is knowledgeable about their treatment(s), symptoms, and concerns and can provide tailored support and resources. This “shared care” model, where oncology and primary care teams both regularly participate in the care of a cancer patient, can help patients feel better supported [13]. While the details of shared care models are not universally agreed upon, there is increasing support for this type of care, from both primary care and oncology care providers [29].

It is important to recognize that this study was only able to capture patient perspectives. While shared care models may benefit patients with cancer, there can be barriers to implementing these types of care models, particular from the perspective of primary care providers. A recent report detailed that, as of September 2022, more than six million Canadians do not have a family doctor [40]. Access to primary care has been an issue in Canada for several years, however the problem was exacerbated by the COVID-19 pandemic, with providers having to manage additional responsibilities [40,41]. While literature suggests that many primary care providers are willing to be involved in a patient’s cancer care, other providers may lack the capacity to commit to a high level of involvement. True “shared care” models may not be possible for all providers, based on individual workloads and competing priorities, however cancer care teams should ensure to send regular updates to primary care regardless, so that all providers have the relevant information for their patients.

### 4.5. Limitations

As the AOPSS sampling criteria are essentially equivalent across provinces, the findings of this study should be largely generalizable to other Canadian settings. However, a few limitations should be acknowledged. Due to the small number of patients who indicated that they did not have a family doctor, we opted to exclude these patients from the analyses, however it would be important to understand how their satisfaction differs from those who indicated they had a family doctor but with little to no involvement. Similarly, the satisfaction of patients who indicated they were unsure about their family doctor’s involvement could provide interesting insights. These analyses are based on patients’ own perceptions of their family doctor’s level of involvement, as there were no actual measures of, for example, how frequently a patient saw their family doctor or how long each visit lasted. We also were unable to understand how long a patient had been attached to their current family doctor, which could also impact patient satisfaction, perhaps particularly in relation to emotional support. While we might expect that patients with a more involved family doctor have a longer-term relationship with that provider, this may not actually be the case, and understanding this distinction could provide relevant insights. Additional questions regarding the details of a patient’s relationship to their primary care provider may be considered for future survey cycles in Alberta.

## 5. Conclusions

Patients may benefit from regular connection and consultation with multiple care providers during their cancer care, not only within their oncology team but also with their PCP or family doctor, to ensure they receive comprehensive and coordinated care. This study found that patients undergoing cancer treatment in CCA were more satisfied with all aspects of their care when their family doctor was involved. Quantifying the positive impacts of family doctor involvement may be an effective way to promote the importance of a “shared care” model of cancer care between ambulatory oncology care teams and primary care. This study would not have been possible without the addition of the unique question about family doctor involvement in the most recent AOPSS cycle in Alberta. Other provinces or jurisdictions may want to consider adding a similar question to their patient experience measures to understand patient satisfaction in this area.

## Figures and Tables

**Table 1:** Distribution of sociodemographic and disease-specific variables by family doctor involvement.

	Family Doctor Involvement				
	Full Sample (*N* = 2204)	Very Involved (*n* = 506)	Somewhat Involved (*n* = 656)	Not Involved (*n* = 817)	*p* ^a^
Age group					0.048
18–39	63 (2.9%)	10 (2.0%)	18 (2.7%)	37 (3.3%)	
60–64	784 (35.6%)	163 (32.2%)	226 (34.5%)	323 (39.5%)	
65–74	805 (36.5)	188 (37.2%)	245 (37.3%)	291 (35.6%)	
75–84	464 (21.1%)	120 (23.7%)	144 (22.0%)	153 (18.7%)	
85 and above	88 (4.0%)	25 (4.9%)	23 (3.5%)	23 (2.8%)	
Sex					0.455
Female	1212 (55.0%)	269 (53.2%)	365 (55.6%)	463 (56.7%)	
Male	992 (45.0%)	237 (46.8%)	291 (44.4%)	354 (43.3%)	
Educational level					0.000
High school or less	888 (40.3%)	242 (47.8%)	280 (42.7%)	290 (35.5%)	
College, trade or technical	697 (31.6%)	152 (30.0%)	211 (32.2%)	280 (34.3%)	
University degree or above	488 (22.1%)	89 (17.6%)	142 (21.6%)	216 (26.4%)	
No response	131 (5.9%)	23 (4.5%)	23 (3.5%)	31 (3.8%)	
Tumour groups					0.046
Breast	492 (22.3%)	105 (20.8%)	145 (22.1%)	199 (24.4%)	
Gastrointestinal	322 (14.6%)	83 (16.4%)	94 (14.3%)	110 (13.5%)	
Genitourinary	263 (11.9%)	58 (11.5%)	80 (12.2%)	101 (12.4%)	
Gynecology	115 (5.2%)	23 (4.5%)	34 (5.2%)	44 (5.4%)	
Hematology	581 (26.4%)	120 (23.7%)	186 (28.4%)	210 (25.7%)	
Intrathoracic	238 (10.8%)	76 (15.0%)	70 (10.7%)	72 (8.8%)	
Other ^b^	193 (8.8%)	41 (6.1%)	47 (7.2%)	81 (9.9%)	
Treatment intent					0.040
Curative	976 (44.3%)	233 (44.1%)	277 (42.2%)	395 (48.3%)	
Non-curative	1018 (46.2%)	252 (49.8%)	343 (52.3%)	374 (45.8%)	
No response	210 (9.5%)	31 (6.1%)	36 (5.5%)	48 (5.9%)	
Cancer centre visited					0.003
Tertiary	1273 (57.8%)	267 (52.8%)	364 (55.5%)	504 (61.7%)	
Regional/Community	931 (42.2%)	238 (47.2%)	292 (44.5%)	313 (38.3%)	
Time since diagnosis (in months)					
Mean (SD)	39.7 (54.7)	42.7 (56.0)	41.8 (56.0)	37.1 (51.8)	0.111

a: Significance testing was performed among the three “family doctor involvement” groups only. b: Other included: CNS, endocrine, head & neck, melanoma, non-melanoma skin, other malignant and sarcoma.

**Table 2:** Linear regression analysis summary for family doctor involvement on six domains of person-centred care.

Domain		b ^a^	SE ^b^	95% CI for b	β ^c^	*t*	*p*
Access to Care	Not involved *						
	Somewhat involved	0.063	0.019	−0.003–0.064	0.062	1.77	0.078
	Very involved	0.030	0.017	0.026–0.101	0.116	3.30	0.001
Coordination & Integration of Care	Not involved *						
	Somewhat involved	0.072	0.014	0.044–0.100	0.127	5.02	0.000
	Very involved	0.162	0.016	0.131–0.193	0.264	10.3	0.000
Emotional Support	Not involved *						
	Somewhat involved	0.085	0.018	0.049–0.121	0.129	4.66	0.000
	Very involved	0.175	0.020	0.135–0.214	0.242	8.64	0.000
Information, Communication & Education	Not involved *						
	Somewhat involved	0.064	0.016	0.032–0.095	0.102	3.93	0.000
	Very involved	0.146	0.018	0.111–0.180	0.214	8.21	0.000
Physical Comfort	Not involved *						
	Somewhat involved	0.044	0.026	−0.008–0.096	0.063	1.64	0.101
	Very involved	0.084	0.030	0.024–0.144	0.107	2.77	0.006
Respect for Patient Preferences	Not involved *						
	Somewhat involved	0.046	0.012	0.023–0.069	0.100	3.86	0.000
	Very involved	0.071	0.013	0.046–0.097	0.143	5.47	0.000

a: b: Unstandardized coefficient; b: SE: Standard error; c: β: Standardized coefficient. *: Reference group.

**Table 3:** Ordinal regression analysis summary for family doctor involvement on overall experience question.

Overall Question		b ^a^	SE ^b^	OR	95% CI for OR	Wald χ^2^	*p*
Rate the quality of care	Not involved *	0		1			
	Somewhat involved	0.274	0.108	1.32	1.07–1.62	6.46	0.011
	Very involved	0.835	0.127	2.31	1.80–2.96	43.2	0.000

a: b: Unstandardized coefficient; b: SE: Standard error; *: Reference group.

## Data Availability

The data used in this study are not publicly available, however may be available upon request. Please contact the corresponding author.
